# Calcimimetics and Vascular Calcification

**DOI:** 10.3390/toxins17060297

**Published:** 2025-06-12

**Authors:** Avinash Chandu, Carolt Arana, Juan Daniel Díaz-García, Mario Cozzolino, Paola Ciceri, José-Vicente Torregrosa

**Affiliations:** 1 Nephrology and Renal Transplant Department, Hospital Clínic, 08036 Barcelona, Spain; achandu@clinic.cat (A.C.); carana@clinic.cat (C.A.); jdiaz@clinc.cat (J.D.D.-G.); vtorre@clinic.cat (J.-V.T.); 2 Department of Health Sciences, University of Milan, 20142 Milan, Italy; paola.ciceri@unimi.it

**Keywords:** vascular calcification, aortic calcification, calcimimetics, cinacalcet, etelecatetide, evocaletide

## Abstract

In patients with chronic kidney disease (CKD), cardiovascular events (CVA) are the main cause of morbidity and mortality. Vascular calcification, linked to bone mineral metabolism disorders such as elevated serum phosphate, parathyroid hormone (PTH), and FGF23, well-known uremic toxins, aggravate this risk. Calcimimetics are allosteric activators of the calcium-sensing receptor (CaSR), a G protein-coupled receptor that regulates PTH secretion and synthesis in response to changes in extracellular calcium in the parathyroid glands. Through direct and indirect mechanisms, they have demonstrated their efficacy in reducing the progression of vascular, valvular, and soft tissue calcification in experimental studies. Although clinical studies in dialysis patients did not achieve statistical significance in their primary objectives, positive results in subgroup analyses suggest that the lack of significance may be attributable to the short follow-up period. This finding highlights the need to consider early treatment strategies, especially in advanced stages of chronic kidney disease, to more effectively address the progression of vascular calcification through serum uremic toxins control.

## 1. Introduction

Cardiovascular diseases represent the primary cause of morbidity and mortality in patients with chronic kidney disease (CKD) [1]. The reduction in estimated Glomerular Filtration Rate (eGFR) serves as an independent and graded predictor of cardiovascular morbidity and mortality, as well as all-cause mortality [2]. Consequently, irrespective of traditional risk factors, moderate CKD (eGFR 30–59 mL/min/1.73 m^2^) and severe CKD (eGFR < 30 mL/min/1.73 m^2^) are classified as high and very high risk, respectively, according to the European Society of Cardiology (ESC/EAS) [3].

These cardiovascular alterations have been associated with disorders of mineral bone metabolism, which are prevalent in advanced stages of CKD, leading to an increase in vascular calcification (VC). This results in elevated arterial stiffness, thereby contributing to increased morbidity and mortality in these patients [1].

The development of calcifications has been linked to several factors, including elevated serum calcium, calcium-phosphorus product, and particularly serum phosphate. The latter positively regulates Pit1, a sodium-dependent phosphate transporter, principal responsible for phosphate uptake in vascular smooth muscle cells. This elevates intracellular levels of inorganic phosphate (Pi) and initiates the process of VC by inducing alterations in smooth muscle cells (SMCs), which undergo a cellular transdifferentiation process involving extracellular vesicles. This process implicates genes related to inducing factors such as Runx2/Cbfa1, alkaline phosphatase, collagen, and osteopontin (OPN), as well as changes in inhibitory factors, including matrix Gla protein (MGP), osteonectin (ON), and OPN [4,5,6]. Additionally, the direct role of PTH in VC has been proposed, as experimental studies have demonstrated that continuous infusion of high PTH concentrations develops calcifications in the aorta and coronary arteries without correlation to elevated phosphorus levels or the degree of renal insufficiency [7].

At present, we have different treatments, such as phosphorus binders, vitamin D analogs, and, most notably, calcimimetics, which have been shown to reduce PTH levels without raising serum calcium and phosphorus contractions. Likewise, their action on structural remodeling at the level of the blood vessel wall has been described, influencing the reduction in VC [8].

## 2. Methods

This is a narrative review that conducted a rigorous PubMed search for “calcimimetics” in combination with the terms “cinacalcet”, “etelecatetide”, “evocalcer”, “calcification”, “vascular calcification”, “aortic calcification,”, or “renal disease”. The search was not limited by language or publication date. A total of 58 publications were identified. Of these, 18 reports provided information on vascular and aortic calcification in the course of randomized controlled trials, 5 were clinical practice studies, and 8 represented isolated case reports. Additional references were found in the reference lists of manuscripts identified in the search. In addition, the EMA and FDA websites were searched for “calcimimetics”.

## 3. History and Evolution of Calcimimetics

Calcimimetics are allosteric activators of CaSR, a G protein-coupled receptor, which regulates PTH secretion and synthesis in response to extracellular calcium changes in the parathyroid glands [9].

Initially, two types of molecules were developed; the first, R-568, had problems with bioavailability, with variable pharmacokinetics, interfering with various drugs through the hepatic cytochrome P-450 pathway [10], so later a second agent was developed, AMG 641 or cinacalcet hydrochloride, this being a more stable molecule [3,11]. In 2004, its commercialization began in the United States and in 2005 in Europe for patients with secondary hyperparathyroidism on dialysis. However, despite good control of biochemical parameters, poor therapeutic adherence was observed, varying between 45.6 and 71%, due to its gastrointestinal side effects [12]. Subsequently, a second-generation calcimimetic, AMG416, known as etecalcetide, was introduced, whose main difference was the route of administration (intravenous), decreasing side effects and can activate CaSR even in calcium-free conditions, being a direct CaSR agonist [13]. Currently under study is a new oral molecule, Evocalcet (MT-4580/KHK7580), which, unlike cinacalcet, has fewer adverse effects related to the digestive tract and requires lower doses than cinacalcet to suppress PTH levels (Table 1) [14,15,16,17].

## 4. Mechanisms of Action: Direct and Indirect

It has been proposed that there are two mechanisms of action, an indirect one in which, following the reduction in serum PTH, the progression of VC would be prevented. This hypothesis was corroborated in an animal model with parathyroidectomised rats (to avoid the influence of calcimimetics on the parathyroid gland), in which, after PTH administration, aortic calcification was induced independently of serum calcium and phosphate levels, suggesting a direct and indirect role of PTH in the induction of VC [7]. Subsequently, another study using the same animal model demonstrated that cinacalcet AMG641 increases urinary calcium and phosphate excretion, leading to reduced serum levels of calcium and phosphate (hypocalcemia and hypophosphatemia). These changes are associated with decreased progression of vascular calcification [18,19,20,21,22].

The second mechanism proposed is direct action due to the fact that CaSR expression is not only limited to the parathyroid gland but is also expressed in different cells such as vascular smooth muscle cells (VSMC), endothelial cells, on the cell surface of phagocytic cells and in monocytes/macrophages, whose activation seems to play an important role in regulating the mineralization of these cells [23,24,25,26].

CaSR activation by calcimimetics enhances CaSR expression by promoting CaSR maturation and transport to the plasma membrane, reduces extracellular matrix formation and therefore decreases type I collagen secretion, and increases α-actin expression, decreasing VSMC mineralization [18]. In addition, CaSR activation promotes phagocytosis of mineral deposits at the level of the arterial wall, reducing VC [18,19]. It is also postulated that the effect of calcimimetics is related to the expression of genes associated with VC-inducing factors, observing an inhibition of Runx2, osteocalcin and osteopontin gene expression in nephrectomy (Nx) rats [27] and increasing the expression of MGP mRNA in vascular cells cultured in vitro and in the aortic arterial wall in a uraemic animal model, since this marker, as previously discussed, is one of the main inhibitors of VC [28].

## 5. Clinical Implication of Calcimimetics in the Reduction in Vascular Calcification

With the introduction of calcimimetics in CKD patients on dialysis, a significant reduction in serum levels of PTH has been observed, and these, in turn, inhibit the mobilization of calcium and phosphate from bone [8]. It is postulated that this effect may contribute to the reduction in VC [27]. Various studies with animal models have observed this relationship. Kawata et al. described that treatment with Cinacalcet at different doses (5 mg/kg and 15 mg/kg) inhibited aortic arterial and aortic valve calcification in rats that underwent Nx associated with a high phosphorus and lactose diet to generate a procalcifying environment. The same results were observed when rats underwent parathyroidectomies [27]. Koleganova et al. A reduction in calcium deposits in the aortic midwall was observed in animals exposed to R-568 compared to the group exposed to calcitriol treatment [29].

On the other hand, it has been described that calcimimetics are related to the reduction in vascular remodeling. In studies carried out with animal models of renal damage, a decrease in vascular remodeling markers such as proliferated cellular nuclear antigen (PCNA) and TGF- β 1 in VSMC was observed in rats treated with R-568, also associated with a lower thickness in the intima and less calcification of the vascular wall [29]. Curiously, another work has described that the administration of R-568 delays the progression of atherosclerosis enhanced by CKD; Ivanovski et al. observed in hypercholesterolemic mouse models apo-E -/-, which develop atherosclerotic lesions at the level of the aorta, that calcimimetics were able to reduce the progression of VC in vivo in both the intima and media layers [23].

In addition to the effects at the vascular level, benefits at the bone level have also been described. In a study carried out in animal models with CKD, a decrease in bone volume was observed, accompanied by an increase in the activity of osteoblasts and osteoclasts, changes attributed to high levels of uremia. In this context, the administration of R-568 demonstrated a dose-dependent improvement in bone volume [30]; this effect is described in other works where treatment with AMG 641 prevented the loss of the number of trabeculae and bone mineralization defects [31].

However, this treatment is indicated for patients with CKD who start renal replacement therapy such as dialysis or patients with primary hyperparathyroidism (HPT), and the use of calcimimetics in the predialysis stage is questioned since reducing PTH levels causes a reduction in its phosphaturic effect and thus increases serum phosphorus levels, making it difficult to control hyperphosphatemia in these stages [32]. Moe et al. conducted an experimental study with an animal model for slowly progressing CKD and administered R-568 to one group and R-568 with calcium supplements to another group. It was observed that at week 34, animals treated only with calcimimetics presented higher phosphorus levels, but later, at week 38, these values did not continue to increase. In the group of animals with R-568 and calcium supplementation, phosphorus values had decreased, so the authors initially suggested that hyperphosphatemia and hypocalcemia could be prevented with oral calcium administration in these stages of CKD [30]. However, when the vascular component was evaluated in this animal model, it was observed that treatment with R-568 alone led to a reduction in aortic, aortic valve and myocardial calcification compared to R-568 plus calcium supplementation [30], suggesting that R-568-induced hyperphosphatemia in the early course of CKD does not have negative consequences on arterial calcification, so calcium supplementation to prevent hypocalcemia in clinical practice should be carried out with caution to balance the biochemical and bone benefits against VC. A combination of calcimimetics with phosphorus binders previously described [33] could reduce VC in the early stages of CKD.

In clinical practice, cinacalcet has been a turning point for the treatment of secondary hyperparathyroidism since it reduces plasma PTH levels without increasing serum calcium and phosphorus values, unlike what occurs with vitamin D analogs. On the other hand, the relationship between Cinacalcet and VC has been studied in different studies (Table 2).

## 6. Effect of Cinacalcet on Calciphylaxis Lesions

In a sub-study of the EVOLVE trial [37], whose primary objective originally assessed the effect of cinacalcet on reducing the risk of death or major cardiovascular events in patients with moderate-to-severe secondary hyperparathyroidism undergoing hemodialysis, no significant reduction was found for this primary endpoint. However, when specifically analyzing the effect of calcimimetics on calcific uremic arteriolopathy (CUA), treatment with cinacalcet was observed to reduce the risk of developing this complication by between 69% and 75%. Additionally, female gender, obesity, diabetes mellitus, and use of vitamin K antagonists were identified as risk factors for CUA [38]. The effect of calcimimetics on CUA has also been described in several case reports highlighting that the use of cinacalcet helped in the resolution of skin ulcers in patients with calciphylaxis, and it was suggested that, by presenting rapid biochemical control, it could benefit the control of CUA [39,40,41].

## 7. Effect of Cinacalcet on Arterial Stiffness in SHPT

Calcimimetics also exerts direct effects on the vessel wall, reducing arterial stiffness, as determined by pulse wave velocity (PWV). Administration of cinacalcet at medium doses (30–60 mg/day) is associated with a significant reduction in aortic PWV values after 12 months of follow-up (9.35 ± 1.83 m/sg vs. 8.66 ± 1.86 m/sg; *p* = 0.030), as well as a trend towards a reduction in left ventricular mass index [42]. This effect is due to the direct action of calcimimetics at the vascular level since, in preclinical studies, it has been shown that the expression of CaSR is altered in the context of arterial hypertension and calcimimetics could produce a dose-dependent vasodilatory effect, modulating vascular tone both in normal and pathological conditions (such as in hypertension) [43,44,45,46].

## 8. Vitamin D Analogues, Calcimimetics and Vascular Calcification

Another point of controversy surrounding VC is the role played by vitamin D analogs. Its use has become widespread as a treatment for HPTs in predialysis stages of CKD, aimed mainly at reducing PTH levels; however, it is also related to elevated levels of phosphorus and calcium. Therefore, in different experimental studies in vitro and in animal models, it is evident that calcitriol promotes the development of VC [47,48,49,50,51]. In addition, it was observed that high-dose Cinacalcet therapy decreases calcium deposits in the arterial media layer; however, when the joint administration of calcitriol and cinacalcet therapy did not show a reduction in signs of VC [47].

This differs from the study carried out by López et al. An increase in skeletal calcification is also described in an animal model (uremic rat) when administering calcitriol or paricalcitol, but when simultaneously administering calcimimetics AMG 641, a reduction in soft tissue calcification and aortic mineralization was observed, associated with excellent control of hypertension [24].

Subsequently, Henley et al. performed a new study where they demonstrated the properties of calcimimetic AMG 641, preventing PTH elevations, parathyroid gland hyperplasia, VC, and renal osteodystrophy in uremic rats treated with adenine. In contrast, when calcitriol was administered at the minimum effective dose to reduce PTH levels (10 ng), it was observed that it exacerbated VC by 40% [31]; however, improvement was observed in bone parameters but not in parathyroid hyperplasia [31].

On the other hand, some preclinical studies suggest that treatment with calcitriol may inhibit VC [52,53]. This disparity in results may be due to the difference in the administered dose of vitamin D analogs in each study. In addition, a lower degree of VC has been described with the use of paricalcitol [54].

However, in a recent experimental study performed in an animal model of CKD, calcitriol was administered at different doses (20 ng/kg and 80 ng/kg), and an increase in VC and severe hemodynamic alterations were observed. Although MGP, a vitamin K-dependent protein that inhibits VC, was increased in this model, treatment with calcitriol was also associated with the upregulation of procalcifying genes in the thoracic aorta such as Pit-1 and Runx2, and this effect was not compensated with a diet rich in vitamin K [55]. Therefore, this association between VC and calcitriol is not only related to elevated serum calcium and phosphorus levels but also has a direct effect on the regulation of procalcifying genes in vascular tissue, causing changes in the VSMC phenotype [4,29,50,56]. In turn, calcitriol suppresses proteins related to PTH/PTHrP, a factor that inhibits the expression of alkaline phosphatase, degrading pyrophosphate, the main inhibitor of VC [57,58].

## 9. Conclusions

In summary, calcimimetics are effective therapeutic agents for the treatment of HPTs. Experimental studies with animal models have demonstrated their role in slowing the progression of vascular, valvular, and soft tissue calcification, associated with their direct effect at the vascular level related to CaSR and regulation of gene expression of VC inhibitory factors, and their indirect effect with the adequate control of PTH, calcium, and phosphorus.

On the other hand, it is important to consider whether interventions to prevent the progression of VC in patients with advanced CKD are being implemented too late, so the objective would be to intervene at earlier stages by developing strategies for early treatment.

## 10. Key Point

1. Cardiovascular disorders are the main cause of morbidity and mortality in patients with chronic kidney disease (CKD) and are related to disorders of bone mineral metabolism that lead to an increase in vascular calcification (VC).

2. Calcimimetics, such as cinacalcet, act as allosteric activators of the calcium-sensing receptor (CaSR), reducing PTH levels without elevating serum calcium and phosphorus concentrations, which may contribute to the reduction in VC.

3. The mechanism of action of calcimimetics in preventing VC appears to be both indirect (through PTH reduction) and direct (acting on vascular smooth muscle cells and other cell types expressing CaSR).

4. Studies in animal models have shown that calcimimetics can inhibit aortic and valvular arterial calcification, reduce vascular remodeling, and improve bone volume in CKD conditions.

5. There is controversy regarding the role of vitamin D analogs in VC, as some studies show that they can promote it, while others suggest an inhibitory effect. The combination of calcimimetics with low doses of vitamin D analogs could be beneficial in reducing the progression of coronary artery calcification in dialysis patients with secondary hyperparathyroidism.

## Figures and Tables

**Table 1:** Common elements and differences in calcimimetics.

	Cinacalcet	Evocalcet	Etelcalcetide	Upacicalcet
Drug composition	Phenylalkylamine	Phenylalkylamine	D-amino acid peptide	Non-peptidic
Route of administration	Oral	Oral	Endovenous	Endovenous
Mechanism of action	CaSR + allosteric modulator	CaSR + allosteric modulator	CaSR + allosteric modulator	CaSR + allosteric modulator
Bioavailability	5.1–28.4%	62.7%	100%	100%
Half-life	30–40 h	20–22 h	18–19 h	1–2 h
Effective dose	30–180 mg/day	1–8 mg/day	2.5–15 mg 3 times a week	25–300 μg 3 times a week
Gastrointestinal adverse effects	30%	15%	15%	1%
Severe hypocalcemia	2.3%	2%	5%	0–2%
CYP2D6 inhibition	High	Low	No	No

Data obtained from the EMA (European Medicines Agency) and FDA (Food and Drug Administration). Abbreviations: CaSR: calcium-sensitive receptor; CYP2D6: cytochrome P450 2D6.

**Table 2:** Clinical studies on the relationship between the use of calcimimetics and vascular calcification.

Clinical Study	Year	Study Design	Population	Primary Endpoint	Results
**ADVANCE** [34]	2011	Randomized Clinical Trial	In total, 360 adult hemodialysis patients with secondary hyperparathyroidism were randomly assigned to treatment with cinacalcet plus low-dose vitamin D or variable doses of vitamin D alone for 52 weeks.	To evaluate the effects of cinacalcet plus low-dose vitamin D on vascular calcification in subjects undergoing hemodialysis over a 52-week period.	Decrease in the total CAC Agatston score from baseline to week 52, being 24% in the cinacalcet group and 31% in the control group, with a treatment difference of −10.3% (*p* = 0.073).
**ADVANCE (post hoc)** [35]	2013	Randomized Clinical Trial	A total of 70 subjects received cinacalcet and low-dose vitamin D, and 120 control subjects received vitamin D sterols.	To evaluate CAC1 progression in patients on cinacalcet and low-dose vitamin D (<2 μg) vs. variable doses of vitamin D.	Reduction in Agatston CAC1 score from 17.8% to 31.3% (*p* = 0.02). Decrease in the progression of aortic valve calcification (*p* = 0.02).
**BONAFIDE** [36]	2014	Multicenter, single-arm clinical trial	Adult patients on dialysis with: - Plasma PTH ≥ 300 pg/mL - Serum calcium ≥ 8.4 mg/dL - Bone-specific alkaline phosphatase > 20.9 ng/mL - Biopsy-proven high-turnover bone disease - On treatment with Cinacalcet	Change in bone formation rate per tissue area (BFR/T.Ar) in patients treated with cinacalcet from baseline to 12-month follow-up.	Bone formation rate/tissue area (BFR/T.Ar) decreased from 728 to 336 μm2/mm2/day, osteoblast perimeter/osteoid perimeter decreased from 17.4 to 13.9%, and eroded perimeter/bone perimeter decreased from 12.7 to 8.3% after 12 months of treatment with cinacalcet (bone biopsies).

Abbreviations: CAC1: coronary artery calcification; BFR/T.Ar2: bone formation rate per tissue area.

## Data Availability

No new data were created or analyzed in this study.
